# A Novel Prebiotic Fibre Blend Supports the Gastrointestinal Health of Senior Dogs

**DOI:** 10.3390/ani13203291

**Published:** 2023-10-21

**Authors:** Melanie Le Bon, Laura Carvell-Miller, Zoe Marshall-Jones, Phillip Watson, Gregory Amos

**Affiliations:** Waltham Petcare Science Institute, Melton Mowbray LE14 4RT, UK; melaniesophie.lebon@gmail.com (M.L.B.); laura.carvell-miller@effem.com (L.C.-M.); phillip.watson@effem.com (P.W.)

**Keywords:** microbiome, dog, canine, gastrointestinal, prebiotic, fibre, senior, sugar beet pulp, cellulose, galacto-oligosaccharide

## Abstract

**Simple Summary:**

Senior pets can suffer from a wide range of age-related diseases that cause distress to the pet and their owner. Diet is one of the easiest ways for improving pet health that can be accessed by pet owners. Fibre is one of the most important ingredients for maintaining gut health. This study aimed to understand whether a novel blend of prebiotic fibres added to a nutritionally complete commercial diet could improve the health of senior dogs. Results show that the prebiotic-blend could improve the gut health of senior dogs as measured by improved faecal quality and beneficial changes to the microbiome. The developed prebiotic fibre blend could have a range of future dietary applications.

**Abstract:**

Senior pets can suffer from a wide range of age-related diseases that can impact the quality of life for the pet and the relationship between a pet and their owner. Dietary fibre plays a key role in shaping the gastrointestinal health in mammalian species. The aim of this study was to investigate the impact of a novel prebiotic fibre blend containing sugar beet pulp, galacto-oligosaccharides, and cellulose on the health of senior dogs when fed on top of a background commercial dry diet. Thirty-two dogs aged >8 years received the prebiotic fibre blend as a dietary topper for 21 days on top of a nutritionally complete diet using a cross-over study design. The prebiotic fibre blend improved the gastrointestinal health of senior dogs as measured through improved faecal quality scores, a reduction in faecal pH, changes to the taxonomic composition of the gut, and a reduction in faecal branched-chain fatty acids, which are markers for proteolytic degradation. Broader systemic measures, such as changes to serum cytokines, were not impacted by the prebiotic fibre blend. In conclusion, a novel prebiotic fibre blend containing sugar beet pulp, galacto-oligosaccharides, and cellulose improved the gastrointestinal health of senior dogs and could have a range of potential future dietary applications.

## 1. Introduction

The gut microbiome plays a pivotal role in many aspects of health in humans and animals [1,2,3]. This includes contribution to efficient digestion and metabolism [4,5], development and regulation of the immune system [6], and synthesis of vitamins and metabolites essential to healthy host functions [7]. Microbiome composition and diversity is influenced and shaped by a wide range of host and environmental factors [8]. The evolution of the microbiome over different life stages has been well characterised in humans, with distinct microbial changes associated with healthy and unhealthy ageing [9]. Recent studies have reported that senior dogs undergo changes similar to that of human inflammation and that gut microbial diversity decreases in ageing dogs [10,11]. Healthy ageing is an important aspect of animal wellbeing, and it is therefore important to consider strategies that can maintain or improve functions of health across the lifespan of a pet. Considering the role of the microbiome in host health, it is important to understand how modulation of the microbiome could lead to improvements in gastrointestinal (GI) health of senior pets. Diet is one of the most common and effective strategies to influence the gut microbiome [12]. A wide range of studies have investigated the impact of diet format (e.g., wet vs. dry), nutrient ratio, and specific ingredients on the microbiome and digestive health of dogs [13,14,15,16]. In particular, dietary fibres are effective ingredients that can be used to predictably change the gut microbiome [16]. Fibres are carbohydrate structures that resist enzymatic digestion by the mammalian host. They can be broadly split into two categories: fermentable fibre, which is fibre that can be readily fermented by the gut microbiota, and non-fermentable fibre, which is fibre that is not readily fermented by the gut microbiota but affects gastrointestinal transit [17,18]. Fibres are important for the production of short-chain fatty acids (SCFAs), which are widely recognised as an important group of metabolites produced by the gut microbiota that play fundamental roles in maintaining intestinal homeostasis, gut barrier function, and regulating the immune system [17,19,20,21]. In dogs, common dietary fibre sources investigated to date include inulin [22,23], beet pulp [24], fructo-oligosaccharides (FOS) [25], and galacto-oligosaccharides (GOS) [26], with varying outcomes on host health. For example, GOS has been associated with improved immunity [26], FOS has been demonstrated to improve apparent total tract digestibility of several minerals [25], and beet pulp has been shown to increase short-chain fatty acid production [24]. Cellulose is commonly used as a fibre source in canine diets, though it is a low-fermentable fibre source [23,27]. However, it can be important for gut homeostasis in mammals and has been shown to prevent gut inflammation in mice [28,29].

Frequently, the impact of highly fermentable fibre sources has been explored in isolation. In this study, we aimed to explore the health benefits to dogs of a mixed prebiotic fibre blend comprising highly fermentable sugar beet pulp, GOS, and low-fermentable cellulose. By measuring a range of both GI specific and wider host measures, we improve our understanding of how to modulate the microbiome and improve GI health in senior dogs using mixed prebiotic fibre supplementation.

## 2. Materials and Methods

### 2.1. Animal and Experimental Design

Thirty-two senior dogs housed at the Pet Health and Nutrition Center (PHNC), Lewisburg, OH, USA, participated in the study. Senior age was defined as >8 years old (Table S1). Three breeds were represented: the Beagle (14 dogs), Mean Average Age 11.1 Years Old +/− 0.5, Body Condition Score 4.8 +/− 0.2, Mean Average Weight 10.7 kg +/− 0.9; Brittany (8 dogs), Mean Average Age 10.2 Years Old +/− 0.8, Body Condition Score 4.8 +/− 0.3, Mean Average Weight 14.1 kg +/− 1.46; and Labrador Retriever (10 dogs), Mean Average Age 11.3 Years Old +/− 0.1, Body Condition Score 5.0 +/− 0.0, Mean Average Weight 27.9 kg +/− 2.3 kg.

The study was conducted using a two-way cross-over design, with dogs split into two experimental groups: A and B. A stratified randomisation approach was used to prevent imbalance between groups and considered the following factors in order of preference: breed, unit, litter, and sex. All dogs were fed Royal Canin Medium (Beagles and Brittany) and Royal Canin Large (Labrador Retriever) the week prior to the study, with this being rotated weekly with IAMS Mature Adult in the weeks preceding this. Dogs in experimental group A were fed a control diet and a novel prebiotic fibre blend as a topper in phase 1, followed by control diet only in phase 2. Dogs in experimental group B received the control diet in phase 1, followed by control diet and a novel prebiotic fibre blend as a topper in phase two. Each phase lasted 21 days with no wash out period between phases.

All animals enrolled in the study were neutered and housed in pen pairs in a free-living environment with indoor/outdoor access during the day (weather permitting). Dogs in different study groups were not housed together in pen-pairs. Rooms were fitted with environmental enrichment and all dogs had daily social human interaction: exercise, grooming, training and play with toys. Dogs were deemed healthy by a veterinarian at the start of the study with no evidence of systemic disease, e.g., arthritis, diabetes, thyroid disorder, liver, or renal impairment requiring treatment. Routine housing, husbandry and exercise regimes were maintained throughout the course of the study. Water was provided ad libitum at all times. The general health and overall condition of each animal were monitored daily by the animal care staff. The exclusion criteria for dogs enrolled in the study were antibiotic use within the 8 weeks prior to the start of the study, prebiotic or probiotic supplementation within the 5 weeks prior to the start of the study, and/or vaccination during or within 14 days prior to the start of the study. Flea/tick and heartworm preventative care was given on Day 4 of each study phase, after faecal collections had been completed from the start of each feeding phase.

The study was reviewed and approved by the WALTHAM Animal Welfare and Ethical Review Body (AWERB) and the Institutional Animal Care and Use Committee (IACUC) at PHNC and followed the general principles and recommendations set out in the ARRIVE guidelines [30].

### 2.2. Diet Formulation

All dogs were fed Royal Canin Indoor Small Breed Adult Dog Dry Dog Food, a complete and balanced kibble-based commercial pet food (Royal Canin, Aimargues, France) that includes FOS and beet pulp as fibre sources. During the topper phase, dogs received the prebiotic fibre blend, consisting of sugar beet pulp, GOS, and cellulose, incorporated into wet pet food (Pedigree, Mars) twice a day before routine mealtime. The prebiotic fibre blend dosage was ~7.5–8.0 g for small-sized dogs, 9.0–9.5 g for medium-sized dogs, and 17.0–17.5 g for large-sized dogs. The ratio of prebiotics was approximately 2:7:1 for sugar beet pulp:cellulose:GOS, respectively. In the control phases, dogs received the same amount of wet diet without inclusion of the prebiotic fibre blend. The diet dosage followed the manufacturer’s recommendation and was calculated according to dog bodyweight categories: small (5–10 kg), medium (10–25 kg) and large (25–40 kg).

The ingredient list for Royal Canin Indoor Small Breed Adult Dry Dog Food at the time of this study was as follows: Brewers Rice, Chicken By-Product Meal, Corn, Brown Rice, Chicken Fat, Wheat Gluten, Natural Flavors, Dried Plain Beet Pulp, Vegetable Oil, Pea Fiber, Fish Oil, Sodium Silico Aluminate, Salt, Potassium Chloride, L-Tyrosine, Calcium Sulphate, Calcium Carbonate, Dl-Methionine, L-Lysine, Fructooligosaccharides, Sodium Tripolyphosphate, L-Arginine, Choline Chloride, Vitamins [Dl-Alpha Tocopherol Acetate (Source Of Vitamin E), L-Ascorbyl-2-Polyphosphate (Source Of Vitamin C), Biotin, D-Calcium Pantothenate, Vitamin A Acetate, Pyridoxine Hydrochloride (Vitamin B6), Niacin Supplement, Folic Acid, Thiamine Mononitrate (Vitamin B1), Vitamin B12 Supplement, Riboflavin Supplement, Vitamin D3 Supplement], Magnesium Oxide, Trace Minerals [Zinc Proteinate, Zinc Oxide, Ferrous Sulphate, Manganese Proteinate, Manganous Oxide, Copper Sulphate, Calcium Iodate, Sodium Selenite, Copper Proteinate], Taurine, L-Carnitine, Rosemary Extract, Preserved With Mixed Tocopherols And Citric Acid. The crude fibre content was 3.7%.

GOS was sourced from Dairy Crest (Dairy Crest Ltd., Claygate House, Littleworth Road, Esher, Surrey, KT10 9PN, UK) and was supplied in a dry powder format. Cellulose and sugar beet pulp were supplied from The Peterson Company (The Peterson Company, Kalamazoo, MI, USA). Cellulose was supplied as dry pellets. Prior to inclusion in the wet diet, cellulose pellets were ground in a food processor for 5 min. Sugar beet pulp was also supplied as dry pellets and prior to inclusion in the wet were soaked for 8 h in deionised water (DI) in an air-tight glass container at 4 °C. For soaking, 250 mL of DI water was used per 100 g of sugar beet pulp. Soaked sugar beet pulp was utilised within 72 h. The sugar beet pulp final mass was adjusted to account for the DI water content.

### 2.3. Sample Collection

Overnight faeces were scored daily for each pen according to the WALTHAM scoring system [31]. Faeces with a score of ≤1.5 or ≥3.75 or containing abnormalities such as mucous or blood were classed as poor and the proportion of poor faeces in relation to the total number of faeces collected was calculated. The total faecal weight per pen was also measured daily.

On days 18–20 of each phase, two freshly voided faecal samples were collected from each dog, at least 12 h apart. Fresh faecal samples were used to measure the pH at a depth of 3 cm using the faecal pH meter Mettler Toledo FiveGo F2 (Mettler Toledo, Columbus, OH, USA) as per the manufacturer’s instructions. Two 200 mg (±10%) aliquots were collected for microbiome analysis and one 1 g (±10%) aliquot was collected for SCFA analysis. All faecal sample aliquots were stored at −80 °C within one hour of collection.

All Labrador Retrievers underwent blood sampling on day 22 of each phase. Blood was collected via jugular blood sampling with 6 mL of blood drawn from each dog.

### 2.4. Haematology, Cytokine, and SCFA Analysis

Complete blood count (CBC) and biochemistry analysis of sera was conducted by IDEXX (Norcross, GA, USA). Cytokines were analysed from sera using the Merck MILLIPLEX^®^ Canine Cytokine multiplex immunoassay following the manufacturer’s instructions (Merck, Darmstadt, Germany). The cytokines tested included GM-CSF, IL-2, IL-6, IL-7, IL-15, IP-10, KC, IL-10, and IL-18. SCFA analysis was conducted by LiveLab (LiveLab ltd, Oakham, UK). In brief, SCFAs were extracted from faecal samples using steam distillation and analysed on an Agilent 7890 GC-FID calibrated using certified standards supplied by Sigma-Aldrich (Merck Life Science UK Limited, Gillingham, UK).

### 2.5. 16S rRNA Sequencing and Bioinformatics

DNA was extracted using the QIAamp 96 PowerFecal QIAcube HT Kit (City Labs 2.0, Manchester, UK). Library preparation and amplicon sequencing were performed by Eurofins Genomics (Eurofins, Hamburg, Germany). Amplicons were generated using primers targeting the V3-V4 16S rRNA region (V3V4 -F; 5′-TACGGGAGGCAGCAG-3′ [32]; and V3V4-R; 5′-CCAGGGTATCTAATCC-3′ [33]) and sequenced on an Illumina MiSeq platform (Illumina, San Diego, CA, USA) using v3 chemistry (2 × 300 bp paired-end reads).

DNA sequences were filtered by Q-Score (Q-Score < 30) and length (<50 bases) using Cutadapt. Adapter sequences were trimmed using Cutadapt. Amplicon sequence variants (ASVs) were generated via DADA2 v1.16.0 using post-QC FASTQ files [34]. Paired FASTQ reads were trimmed and then filtered to remove reads containing Ns or with maximum expected errors ≥2. Forward and reverse reads were merged by overlapping sequence and chimeras were removed before taxonomic assignment. ASV taxonomy was assigned to the genus level using the SILVA v.138 database with a minimum bootstrapping support of 50% as previously described using the Ribosomal Database Project (RDP) Classifier [35,36].

### 2.6. Statistical Analysis

All statistical analyses were performed in R version 3.6.3 (29 February 2020), The R Foundation for Statistical Computing. Statistical significance was accepted at unadjusted *p* ≤ 0.05 unless otherwise stated.

Haematology, SCFA, and cytokine analysis: For analysis of the haematology faecal pH, SCFA, and cytokine data, each variable was modelled as the response in a linear mixed effects model, with prebiotic fibre blend treatment as the fixed effect, and individual animal and phase as the random effect. Model residuals were visually assessed to see if they violated model assumptions by plotting the residuals against the fitted values and normal probability plot. Log transformation was applied where necessary to reduce heteroscedasticity.

Faecal quality: Analysis of faecal quality was analysed using a faecal score and faecal weight recorded daily during the trial at the pen level. For the faecal score and faecal weight, linear mixed effects models were fit modelling the response against diet as the fixed effect with day in phase nested in pen pair as the random structure.

Faecal pH data were modelled as the response in a linear mixed effects model, with prebiotic fibre blend treatment as the fixed effect, and the individual animal and phase as the random effect. Model residuals were visually assessed to see if they violated model assumptions by plotting the residuals against the fitted values and normal probability plot. Log transformation was applied where necessary to reduce heteroscedasticity.

For proportion of poor faeces, binomial generalised linear mixed effects models were fit modelling the binary response (acceptable = 0, poor = 1) against diet as the fixed effect and with day in phase nested in pen pair as the random structure.

16S rRNA ASV analysis: All 16S rRNA analysis was performed on ASVs. Following bioinformatic processing, ASV counts were converted to relative abundance for each sample. ASVs that had fewer than two samples within a treatment group with non-zero counts or a relative abundance <0.0001 were filtered and removed.

Shannon diversity was estimated on filtered data using the formula:

$$H=-\sum_{j=1}^{S}p_{i}\ln p_{i}$$

Shannon diversity was fitted to a linear mixed effect model with treatment as the fixed effect, sample set nested in individual animal nested in pen pair and phase as the random effect to account for technical replicates. The contrast between the treatment groups’ estimated means was calculated.

The filtered relative abundance was pseudo logit transformed (logit(count + 2/total + 4)) and used to explore community diversity between treatment group with a Bray–Curtis nMDS and displayed 95% confidence ellipses. Permutational multivariate analysis of variance (PERMANOVA) was performed on the Bray–Curtis distances using the phyloseq and vegan package. Permutational analysis for the multivariate homogeneity of dispersions (PERMDISP2) was conducted to assess the homogeneity of dispersion between the diets.

PLS-DA was applied to the filtered logit alternative dataset, with diet as the response variable. The number of components was estimated using 5-fold cross validation. Results were presented as a score plot with 95% confidence ellipses coloured by treatment group.

Differential analysis of relative abundance was performed on filtered ASVs using DESeq2 [37]. The model applied in DESeq2 fitted the response against treatment as the fixed effect. Comparisons were made between the treatment groups for all ASVs with statistical significance being defined as Benjamini–Hochberg (BH) adjusted *p*-value ≤ 0.01.

## 3. Results

### 3.1. Supplementation with Prebiotic Fibre Blend Improves Digestive Health

**Figure 1:**
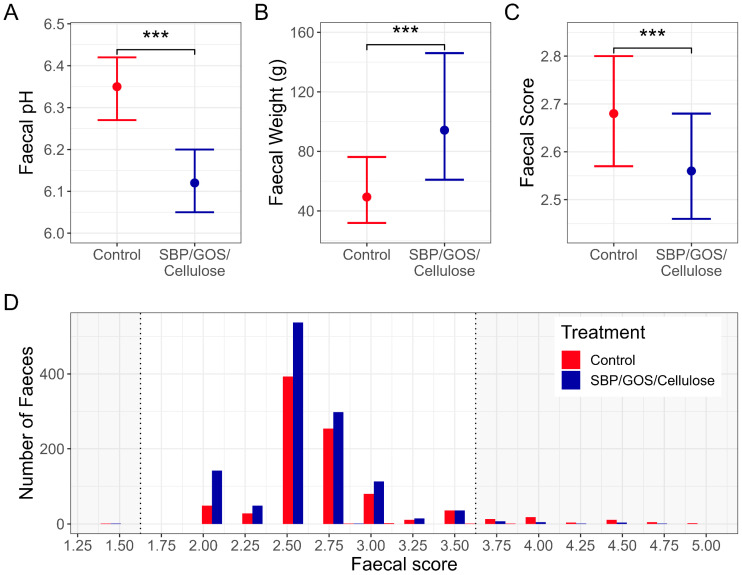
Gastrointestinal health measures for the control diet vs. control diet plus SBP/GOS/cellulose prebiotic fibre blend. (**A**) Faecal pH. (**B**) Mean faecal weight with 95% confidence levels. (**C**) Mean faecal scores mean with 95% confidence levels. (**D**) Distribution of faecal scores. ***: *p* ≤ 0.001.

To understand the impact of the prebiotic fibre blend on canine health, we conducted a feeding study in 32 senior dogs following a two-way crossover study design with two consecutive 21-day feeding phases. Initially, we assessed digestive health using faeces quality. The 17-point WALTHAM scoring stool chart was used where higher scores indicate looser faeces, lower scores indicate harder faeces, and the optimal score are at the mid-point of 2.5 [31]. Faeces with a score of ≤1.5 or ≥3.75 or containing abnormalities such as mucous or blood were classed as poor. Despite a low incidence of poor faeces in the study when using the control diet, the prebiotic fibre blend reduced the incidence of poor faeces further, with a significantly lower proportion of faecal scores classed as poor when dogs received the prebiotic fibre blend (0.3%) compared to the control (1.5%) (*p* ≤ 0.001, Figure 1). Further measures of faecal quality demonstrated an impact on digestive health by the fibre prebiotic blend, with dogs fed the prebiotic fibre blend having a significant decrease in faecal scores (*p* ≤ 0.001, Figure 1), indicating firmer faeces and an increase in daily faecal weight (*p* ≤ 0.001, Figure 1) (Figure 1). Furthermore, faecal pH was significantly reduced by the prebiotic fibre blend, indicative of increased colonisation resistance to pathogens (*p* ≤ 0.001, Figure 1) [38].

In addition to faecal quality, we conducted a number of assays on collected blood from Labrador Retriever dogs to explore the impact of the prebiotic fibre blend on immunological parameters. There was no significant impact on the serum cytokine population; however, the study was not powered to see an effect for immune measures. All blood measures remained within the healthy range for both treatment groups throughout feeding of both diets (Table S2). Collectively, these data indicate that the prebiotic fibre blend improves digestive function but has no measurable impact on immune measures within the limitations of blood sampling animals for this study.

### 3.2. Supplementation with Prebiotic Fibre Blend Changes the Microbiome and Decreases Abundance of Branch-Chain Fatty Acids

**Figure 2:**
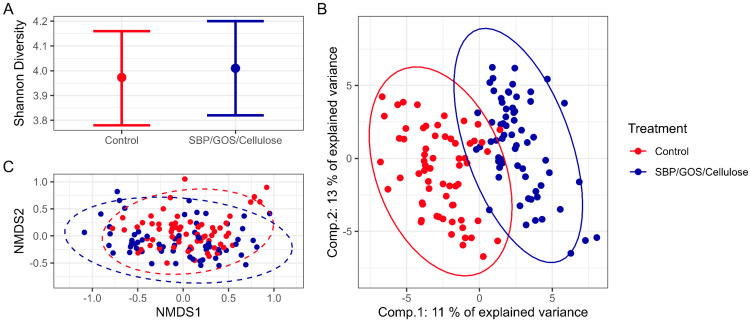
Microbiome diversity analysis for the control diet vs. control diet plus SBP/GOS/cellulose prebiotic fibre blend. (**A**) Shannon diversity index mean and 95% confidence levels. (**B**) Bray–Curtis nMDS plot. (**C**) PLS-DA plot.

We next investigated the impact of the prebiotic fibre blend on the microbiome. Collected faeces were analysed for taxonomic composition using 16S rRNA sequencing. In total, 354 unique ASVs were detected across all samples. No impact was observed on the alpha-diversity of the microbiome following supplementation with fibre and prebiotic blend as measured through the Shannon diversity index. However, there was a change in the community composition, with the visualisation of Bray–Curtis dissimilarity measures using a non-metric dimensional scaling (nMDS) plot demonstrating some separation of groups (Figure 2). The difference between diet groups is supported by PERMANOVA with *p* ≤ 0.001, although with a very low R2 (0.04), implying that a considerable amount of variation in the data is not related to diet. Analysis of the homogeneity of group dispersions, using the PERMDISP2 procedure, indicates a significant difference in dispersion between the diet groups with *p* = 0.036. A partial least-squares discriminant analysis (PLS-DA) further demonstrated that the microbial composition could be separated according to diet fed (Figure 2).

**Figure 3:**
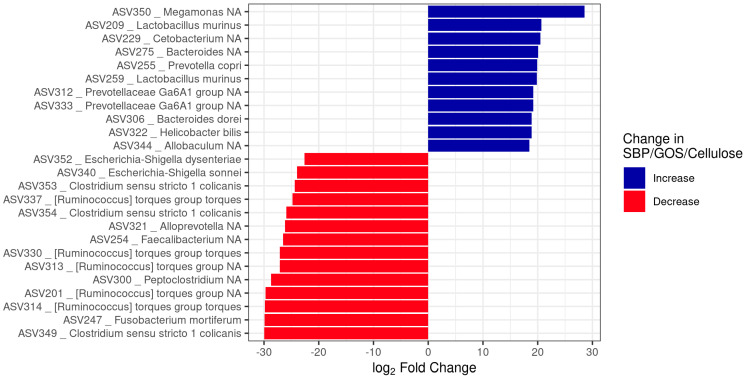
Taxonomic changes driven by prebiotic fibre blend. Top 25 taxa (based on absolute Log2FC) with differential abundance between control and SBP/GOS/cellulose identified by DESeq2 analysis (BH adjusted *p*-value ≤ 0.01).

Investigation into the impact of the fibre and prebiotic blend on the abundance of individual bacterial taxa was conducted using DESeq2. Regardless of diet, the top five most abundant families were *Fusobacteriaceae*, *Prevotellaceae*, *Peptostreptococcaceae*, *Bacteroidaceae*, and *Lactobacillaceae* (Figure 3). However, a range of taxa were increased or decreased depending on diet supplementation, further supporting that the prebiotic fibre blend changes aspects of the microbiome composition (Figure 3). Analysis for the top 25 ASVs ranked by absolute log2-FC with BH adjusted *p*-value ≤ 0.01 demonstrated that the taxa increased by the prebiotic fibre blend relative to the control were classified as the genera *Megamonas*, *Cetobacterium*, *Bacteroides*, *Prevotella*, *Lactobacillus*, *Bacteroides*, *Helicobacter*, and *Allobaculum*. ASVs that were decreased by the prebiotic fibre blend relative to the control were classified as the genera *Escherichia*, *Clostridium*, *Alloprevotella*, *Ruminococcus*, *Peptoclostridium*, *Faecalibacterium*, and *Fusobacterium*. Notably, two ASVs that were significantly reduced by the prebiotic fibre blend were classified as *Escherichia*/*Shigella*, *Peptoclostridium* and are genera containing well-known pathogens, whereas acid-tolerant *Helicobacter* was increased by the prebiotic fibre blend.

To investigate the potential impact of the microbiome changes on host health, we next assessed the profile of short-chain fatty acid (SCFA) profiles (Table 1). Common markers for proteolytic degradation, branched chain fatty acids (BCFAs), were reduced in abundance, with a significant reduction in iso-butyric acid (*p* < 0.05) and total BCFA (*p* < 0.05). Despite the marked reduction in faecal pH, the overall increase observed in short-chain fatty acids was not statistically significant when feeding the prebiotic fibre blend.

## 4. Discussion

The aim of this study was to investigate the impact of a novel prebiotic fibre blend containing sugar beet pulp, cellulose, and GOS on the health of senior dogs when fed on a background commercial dry diet. Although the background commercial diet contained fibre sources, including FOS and sugar beet pulp, this study demonstrates the benefits of an additional high- and low-fermentable prebiotic mix. Over a range of analyses, we demonstrate that the blend significantly improved GI health as measured through faecal quality, faecal pH, changes to the taxonomic composition of the gut, and reduction in branched chain fatty acids. Collectively, these measures show a positive impact on the gut health of senior dogs by the prebiotic blend used in this study.

Although the overall incidence of loose stools was low during the study, there was a significant decrease in the number of incidences when dogs were fed the prebiotic fibre blend. Considering loose stools are a serious concern of pet owners, this prebiotic blend could serve as a supportive intervention to improve faecal quality. An increase in fibre is widely known to improve faecal consistency and can likely be attributed to physical characteristics of fibre such as water retention but also changes in the microbial population [39]. Further work would be needed in populations predisposed to, or with higher incidences of, diarrhoea to confirm its efficacy in this regard.

In addition to the observed effects on faecal quality, there were also a number of changes in the microbiome, with changes in the microbiota composition demonstrated despite there being no impact on overall species alpha-diversity. The results suggest that the proposed prebiotic-fibre blend promoted the growth of beneficial bacteria. For example, two *Lactobacillus* ASVs were increased by the prebiotic fibre blend. *Lactobacillus* is typically associated with a wide range of beneficial host effects through mechanisms such as immunomodulation, epithelial cell binding, carbohydrate metabolism, and GABA production [40,41,42]. There are numerous commercially available probiotic products containing *Lactobacillus* species, marketed to promote host benefit; however, here, we show that such bacterial species already present in the gut can be elevated via the feeding of prebiotic blends. This latter approach has some benefits since *Lactobacillus* probiotics can be technically challenging to incorporate into food products [43].

There was a significant reduction in the abundance of two taxa associated with pathogens in the prebiotic fibre group with significant reductions in *Escherichia*/*Shigella* and *Peptoclostridium*. This could be explained by the reduction in faecal pH observed with the prebiotic fibre blend, a response that enhances GI colonisation resistance [38]. Notably, there was an increase in *Helicobacter* in the fibre-prebiotic group, a genus able to survive harsh acidic conditions. Several studies have shown non-*Helicobacter pylori Helicobacter* species to be widespread in clinically healthy dogs, with estimates ranging from 67 to 86% [44].

*Megamonas* and *Cetobacterium* were also strongly influenced by the prebiotic blend. *Megamonas* has been demonstrated to be increased by common prebiotics such as inulin and fructooligosaccharides, with *Meganomonas* being a taxon that is amongst the most prevalent in healthy dogs [22]. However, along with *Cetobacterium*, the role of these organisms in canine health is unknown, demonstrating the need for more research to elucidate their role in canine health.

Accompanying changes to the microbiota were changes to microbial metabolite production, specifically BCFAs. BCFAs are a marker for proteolytic degradation, a process that is known to produce a range of potentially toxic metabolites including phenolic compounds, amines, and volatile sulphur compounds [45,46]. This result suggests that the fibre prebiotic blend leads to a reduction in proteolytic degradation and its associated toxic metabolites, potentially through modulation of the microbiome. This result is consistent with previous findings showing a decrease in isovaleric acid by GOS supplementation in dogs [26]. Despite an observed decrease in *Faecalibacterium* by the prebiotic and fibre blend, there was no significant change in overall SCFA abundance measured in the faeces. This was surprising considering the well-established role of Faecalibacterium in SCFA production in humans and suggests functional redundancy in the microbiome and highlights the need for further canine-specific research on the role of microbes in host health. A clear limitation of this analysis is the measurement of SCFA in total faeces does not take into account potentially increased or decreased absorption by the intestinal epithelial cells.

To investigate the wider systemic effects of the prebiotic blend, blood measurements were also taken; however, there was no evidence of any impact of the fibre and prebiotic blend on wider systemic host measures or on several aspects of immunity. Considering key immune-modulatory short-chain fatty acids such as butyrate or propionate were not increased, this is perhaps not surprising, and indicates in the short-term, the benefits of the fibre and prebiotic blend are limited to the digestive function. Future work in this area should ensure appropriate powering for immune measures, as this was a limitation in the current study.

## 5. Conclusions

In conclusion, this study demonstrated the beneficial impact of a novel prebiotic fibre blend on markers of GI health in healthy senior dogs, including the faecal quality, pH, microbial metabolite profile, and microbiota composition when fed on top of a background commercial diet. No wider systemic benefits, such as immunity, were identified. However, investigating the efficacy of this combination in dogs predisposed to GI issues or in a clinical cohort suffering from chronic or acute GI disorders could represent a fruitful future avenue of investigation.

## Figures and Tables

**Table 1:** Faecal short-chain fatty acid analysis * Log-transformed and fold change reported.

Concentration (mg/g)	Control (95% CI)	SBP/GOS/Cellulose (95% CI)	Difference in Means (95% CI)	Unadjusted *p*-Value
Acetic Acid	1636.2 (1411.1, 1861.2)	1813.2 (1588.2, 2038.2)	−177.0 (−432.8, 78.8)	0.175
Propionic Acid	1320.5 (1165.9, 1475.2)	1255.1 (1100.4, 1409.7)	65.5 (−151.2, 282.1)	0.554
Butyric Acid *	789.9 (666.9, 935.7)	846.7 (714.8, 1003.0)	0.9 (0.77, 1.13)	0.480
Isobutyric Acid	81.1 (64.3, 97.9)	53.4 (36.6, 70.2)	27.7 (5.6, 49.8)	**0.014**
Valeric Acid	125.4 (60.1, 190.6)	161.1 (95.82, 226.3)	−35.7 (−102.5, 31.1)	0.295
Isovaleric Acid	354.5 (311.9, 397.1)	297.4 (254.7, 340.0)	57.1 (−3.2, 117.4)	0.063
Lactic Acid	268.6 (240.0, 297.1)	255.0 (226.4, 283.5)	13.6 (−3.7, 30.8)	0.122
Total SCFA	3997.4 (3519.5, 4475.3)	4151.7 (3673.7, 4629.6)	−154.3 (−746.5, 438.0)	0.610
Total BCFA *	400.6 (346.6, 463.1)	322.4 (278.9, 372.7)	1.2 (1.0, 1.5)	**0.040**
Ratio SCFA/BCFA *	9.2 (8.2, 10.3)	12.3 (11.0, 13.8)	0.7 (0.6, 0.9)	**<0.001**

## Data Availability

The data presented in this study are available on request from the corresponding author.

## Supplementary Materials

The following supporting information can be downloaded at: https://www.mdpi.com/article/10.3390/ani13203291/s1, Table S1. Study animal details; Table S2. Blood haematology and biochemistry results; Table S3. Serum cytokine concentrations.
