# Supplementary material for: A Novel Prebiotic Fibre Blend Supports the Gastrointestinal Health of Senior Dogs
Source: Animals (Basel). 2023 Oct 21;13(20):3291. doi: 10.3390/ani13203291 (PMC10603684; doi:10.3390/ani13203291)
Supplement: Supplementary file 1 [file animals-13-03291-s001.zip › animals-2645126-supplementary.pdf]

Supplementary Information

Table S1. Study animal details.

AnimalID	Timepoint	DateofBirth	Breed	Diet	BodyWeight	Sex	NeuterStatus	Faecal pH	Shannon Diversity
C06-0031	Phase 1	01/07/2006	Beagle	SBP/GOS/Cellulose	15100	F	N	6.02	3.85
C06-0031	Phase 2	01/07/2006	Beagle	Control	15100	F	N	6.45	3.99
C07-0011	Phase 1	20/06/2007	Beagle	SBP/GOS/Cellulose	11100	M	N	6.4	3.39
C07-0011	Phase 2	20/06/2007	Beagle	Control	11100	M	N	6.14	3.99
C07-0016	Phase 1	20/06/2007	Beagle	Control	10900	F	N	5.94	4.25
C07-0016	Phase 2	20/06/2007	Beagle	SBP/GOS/Cellulose	10900	F	N	6.45	3.97
C07-0017	Phase 1	20/06/2007	Beagle	SBP/GOS/Cellulose	11200	M	N	6.29	3.92
C07-0017	Phase 2	20/06/2007	Beagle	Control	11200	M	N	6.7	4.12
C07-0033	Phase 1	29/06/2007	Beagle	SBP/GOS/Cellulose	10400	M	N	5.6	3.5
C07-0033	Phase 2	29/06/2007	Beagle	Control	10400	M	N	6.31	3.41
C07-0034	Phase 1	29/06/2007	Beagle	SBP/GOS/Cellulose	7700	F	N	6.18	3.73
C07-0034	Phase 2	29/06/2007	Beagle	Control	7700	F	N	6.52	3.7
C07-0035	Phase 1	29/06/2007	Beagle	SBP/GOS/Cellulose	9700	F	N	6.23	3.85
C07-0035	Phase 2	29/06/2007	Beagle	Control	9700	F	N	6.21	4.12
C07-0048	Phase 1	19/09/2007	Labrador Retriever	Control	23400	F	N	6.21	4.3
C07-0048	Phase 2	19/09/2007	Labrador Retriever	SBP/GOS/Cellulose	23400	F	N	5.99	4.23
C07-0051	Phase 1	04/10/2007	Labrador Retriever	SBP/GOS/Cellulose	34300	M	N	6.02	4.14
C07-0051	Phase 2	04/10/2007	Labrador Retriever	Control	34300	M	N	5.93	3.78
C07-0057	Phase 1	04/12/2007	Labrador Retriever	SBP/GOS/Cellulose	27400	F	N	6.11	4.68
C07-0057	Phase 2	04/12/2007	Labrador Retriever	Control	27400	F	N	6.09	4.69
C07-0059	Phase 1	04/12/2007	Labrador Retriever	SBP/GOS/Cellulose	27600	M	N	6.6	3.98
C07-0059	Phase 2	04/12/2007	Labrador Retriever	Control	27600	M	N	6.29	4.55
C08-0002	Phase 1	05/01/2008	Labrador Retriever	Control	28000	M	N	6.11	4.23
C08-0002	Phase 2	05/01/2008	Labrador Retriever	SBP/GOS/Cellulose	28000	M	N	5.77	4.43
C08-0003	Phase 1	05/01/2008	Labrador Retriever	Control	30600	M	N	6.42	4.42
C08-0003	Phase 2	05/01/2008	Labrador Retriever	SBP/GOS/Cellulose	30600	M	N	5.59	4.29
C08-0013	Phase 1	25/02/2008	Labrador Retriever	Control	22600	F	N	6.17	3.67
C08-0013	Phase 2	25/02/2008	Labrador Retriever	SBP/GOS/Cellulose	22600	F	N	6.34	4.31
C08-0014	Phase 1	25/02/2008	Labrador Retriever	Control	29300	M	N	6.4	3.89
C08-0014	Phase 2	25/02/2008	Labrador Retriever	SBP/GOS/Cellulose	29300	M	N	6.74	4.1
C08-0016	Phase 1	27/02/2008	Labrador Retriever	SBP/GOS/Cellulose	31400	M	N	6.05	4.15
C08-0016	Phase 2	27/02/2008	Labrador Retriever	Control	31400	M	N	6.55	4.29
C08-0019	Phase 1	15/05/2008	Brittany	Control	17800	M	N	6.05	4.5
C08-0019	Phase 2	15/05/2008	Brittany	SBP/GOS/Cellulose	17800	M	N	6.01	4.18
C08-0020	Phase 1	15/05/2008	Brittany	SBP/GOS/Cellulose	15000	M	N	6.15	4.15
C08-0020	Phase 2	15/05/2008	Brittany	Control	15000	M	N	6.47	3.98
C08-0021	Phase 1	15/05/2008	Brittany	SBP/GOS/Cellulose	9800	F	N	6.11	4.26
C08-0021	Phase 2	15/05/2008	Brittany	Control	9800	F	N	6.46	4.18
C08-0025	Phase 1	15/05/2008	Brittany	SBP/GOS/Cellulose	14400	F	N	5.96	4.15

C08-0025	Phase 2	15/05/2008	Brittany	Control	14400	F	N	6.65	4.17
C08-0026	Phase 1	15/05/2008	Brittany	Control	15100	M	N	6.24	4.43
C08-0026	Phase 2	15/05/2008	Brittany	SBP/GOS/Cellulose	15100	M	N	5.97	4.23
C08-0046	Phase 1	05/07/2008	Labrador Retriever	SBP/GOS/Cellulose	23900	F	N	6.43	4.51
C08-0046	Phase 2	05/07/2008	Labrador Retriever	Control	23900	F	N	7.11	4.53
C09-0004	Phase 1	13/02/2009	Beagle	SBP/GOS/Cellulose	11100	M	N	5.8	3.46
C09-0004	Phase 2	13/02/2009	Beagle	Control	11100	M	N	6.33	2.6
C09-0005	Phase 1	13/02/2009	Beagle	Control	10200	F	N	6.34	3.58
C09-0005	Phase 2	13/02/2009	Beagle	SBP/GOS/Cellulose	10200	F	N	5.96	3.95
C09-0006	Phase 1	13/02/2009	Beagle	Control	10000	F	N	6.55	3.78
C09-0006	Phase 2	13/02/2009	Beagle	SBP/GOS/Cellulose	10000	F	N	5.91	4.17
C09-0007	Phase 1	13/02/2009	Beagle	SBP/GOS/Cellulose	11300	M	N	6.36	3.53
C09-0007	Phase 2	13/02/2009	Beagle	Control	11300	M	N	6.23	3.65
C09-0008	Phase 1	13/02/2009	Beagle	Control	11300	M	N	6.32	3.87
C09-0008	Phase 2	13/02/2009	Beagle	SBP/GOS/Cellulose	11300	M	N	5.92	4.05
C09-0010	Phase 1	13/02/2009	Beagle	Control	9800	F	N	6.24	3.84
C09-0010	Phase 2	13/02/2009	Beagle	SBP/GOS/Cellulose	9800	F	N	6.02	3.93
C09-0043	Phase 1	08/10/2009	Beagle	Control	9300	F	N	6.11	2.72
C09-0043	Phase 2	08/10/2009	Beagle	SBP/GOS/Cellulose	9300	F	N	6.04	3.37
C10-0018	Phase 1	24/06/2010	Brittany	Control	12900	M	N	6.37	4.39
C10-0018	Phase 2	24/06/2010	Brittany	SBP/GOS/Cellulose	12900	M	N	6.11	4.2
C10-0019	Phase 1	24/06/2010	Brittany	Control	13800	M	N	6.39	4.01
C10-0019	Phase 2	24/06/2010	Brittany	SBP/GOS/Cellulose	13800	M	N	6.62	4.43
C10-0020	Phase 1	24/06/2010	Brittany	Control	13600	M	N	6.34	4.21
C10-0020	Phase 2	24/06/2010	Brittany	SBP/GOS/Cellulose	13600	M	N	6.1	4.08

Table S2. Blood hematology and biochemistry results

Parameter (unit)	*Normal range*	Control		SBP/GOS/Cellulose	
		mean	SD	mean	SD
**RBC (M/µL)**	*5.39-8.7*	6.38	0.37	6.31	0.39
**% Hematocrit**	*38.3-56.5*	45.96	2.11	45.33	2.82
**Hemoglobin (g/dL)**	*13.4-20.7*	14.85	0.86	14.65	0.80
**MCV (fL)**	*59-76*	72.40	1.65	71.80	1.48
**MCH (pg)**	*21.9-26.1*	23.29	0.71	23.25	0.49
**MCHC (g/dL)**	*32.6-39.2*	32.29	0.63	32.34	0.51
**% Reticulocytes**		0.52	0.21	0.57	0.29
**Reticulocytes (K/µL)**	*10-110*	33.30	14.30	35.70	16.84
**Retic HGB (pg)**	*22.3-29.6*	26.15	0.96	26.47	1.07
**WBC (K/µL)**	*4.9-17.6*	8.08	1.42	7.91	1.34
**% Neutrophils**		60.92	5.85	61.18	5.00
**% Lymphocytes**		26.12	6.10	25.06	5.70
**% Monocytes**		7.89	1.47	8.59	1.89
**% Eosinophils**		4.93	1.64	5.00	1.65
**% Basophils**		0.14	0.10	0.17	0.07
**Neutrophils (K/µL)**	*2.94-12.67*	4.94	1.10	4.84	0.90
**Lymphocytes (K/µL)**	*1.06-4.95*	2.10	0.58	1.97	0.53
**Monocytes (K/µL)**	*0.13-1.15*	0.64	0.18	0.69	0.23
**Eosinophils (K/µL)**	*0.07-1.49*	0.65	0.77	0.40	0.14
**Basophils (K/µL)**	*0-0.1*	0.01	0.01	0.01	0.01
**Glucose (mg/dL)**	*63-114*	83.20	18.74	83.90	10.19
**SDMA (µg/dL)**	*0-14*	13.20	3.85	12.20	4.08
**Creatinine (mg/dL)**	*0.5-1.5*	0.83	0.22	0.81	0.20
**BUN (mg/dL)**	*9-31*	13.40	4.53	13.00	3.80
**BUN:Creat Ratio**		16.14	2.50	16.04	1.41
**Phosphorus (mg/dL)**	*2.5-6.1*	4.10	0.75	4.02	0.65
**Calcium (mg/dL)**	*8.4-11.8*	9.48	0.45	9.28	0.37
**Sodium (mmol/L)**	*142-152*	145.90	3.60	145.60	2.76

**Potassium (mmol/L)**	*4.0-5.4*	4.52	0.26	4.44	0.25
**Na:K Ratio**	*28-37*	32.30	1.95	32.80	1.69
**Chloride (mmol/L)**	*108-119*	110.60	3.13	111.30	2.16
**TCO2 (mmol/L)**	*13-27*	21.50	0.97	20.20	1.03
**Anion Gap (mmol/L)**	*11-26*	18.40	1.35	18.50	2.01
**Total Protein (g/dL)**	*5.5-7.5*	5.72	0.43	5.65	0.30
**Albumin (g/dL)**	*2.7-3.9*	2.85	0.15	2.76	0.13
**Globulin (g/dL)**	*2.4-4.0*	2.87	0.47	2.89	0.30
**Alb/Glob**	*0.7-1.5*	1.03	0.19	0.97	0.13
**ALT (U/L)**	*18-121*	66.10	41.33	77.30	65.83
**AST (U/L)**	*16-55*	28.70	6.24	29.00	3.62
**ALP (U/L)**	*5-160*	51.90	27.35	52.10	22.06
**GGT (U/L)**	*0-13*	3.70	0.67	3.70	1.16
**Bili-Total (mg/dL)**	*0.0-0.3*	0.19	0.03	0.20	0.05
**Bili-Unconj (mg/dL)**	*0.0-0.2*	0.09	0.03	0.10	0.05
**Bili-Conj (mg/dL)**	*0.0-0.1*	<0.1		<0.1	
**Cholesterol (mg/dL)**	*131-345*	265.70	51.64	260.80	53.26
**Crea Kinase (U/L)**	*10-200*	89.50	29.98	86.10	25.61

Table S3. Serum cytokine concentrations

Concentration (pg/ml)	Control (95% CI)	SPB/GOS/Cellulose (95% CI)	Difference in means (95% CI)	Unadjusted p-value
**GM-CSF***	54.1 (10.2, 287)	43.2 (8.15, 229)	0.80 (0.58, 1.10)	0.143
**IL-2***	56.1 (7.74, 407)	43.8 (6.0, 318.0)	0.78 (0.55, 1.10)	0.135
**IL-6***	42 (8, 233)	39 (7, 217)	0.93 (0.72, 1.21)	0.549
**IL-7***	71.8 (12.3, 421.0)	58.9 (10.1, 345.0)	0.82 (0.63, 1.08)	0.131
**IL-15***	166 (21, 1308)	116 (14.7, 914)	0.70 (0.41, 1.21)	0.169
**IP-10**	46.2 (34.1, 58.2)	46.7 (34.6, 58.7)	-0.52 (-4.92, 3.88)	0.792
**KC**	155 (81, 230)	158 (83, 233)	-2.4 (-37.1, 32.2)	0.876
**IL-10***	25.1 (7.2, 87.8)	23.3 (6.7, 81.6)	0.93 (0.71, 1.21)	0.548
**IL-18***	142 (30, 685)	124 (26, 596)	0.87 (0.67, 1.14)	0.263

* For: GM-CSF, IL-2, IL-6, IL-7, IL-15, IL-10 and IL-18, log transformation was applied for statistical testing.
